# Catalyzing Chemistry Innovation: How Emerging Technologies Are Transforming the Chemical Industry

**DOI:** 10.1021/acscentsci.5c00530

**Published:** 2025-06-25

**Authors:** F. Gomollón-Bel, J. García-Martínez

## Introduction

Scientific discovery alone is not enough—transforming breakthroughs into real-world solutions requires a clear pathway from innovation to commercialization. This paper explores how emerging technologies in chemistry can catalyze industrial transformation, and how initiatives such as the Top Ten Emerging Technologies in Chemistry by the the International Union of Pure and Applied Chemistry (IUPAC) provide a roadmap to bridge the gap between lab discoveries and market impact. We highlight the crucial role of entrepreneurship, interdisciplinary collaboration, and digital tools in accelerating this transition. We also examine the barriers that prevent many discoveries from reaching commercialization and present IUPAC’s efforts, including the Chemistry Entrepreneurship project, to empower chemists with the tools to innovate beyond the lab.

Chemistry is central to solving global challenges, from the climate crisis to electrification and the energy transition.^1^ With this same spirit, IUPAC envisioned the Top Ten Emerging Technologies in Chemistry initiative in 2019 to highlight ground-breaking advances that could shape and transform the current industrial landscape. This selection underscores the importance of cross-collaboration between public and private ventures, showcasing clever chemical concepts with built-in features such as sustainability and circularity. Aligned with the United Nations’ Sustainable Development Goals (SDGs), each year IUPAC experts select a series of ideas to spark new innovations in chemistry, materials science, energy, and healthcare, which could convey creative solutions to the current crises.^2^

In this sense, IUPAC not only organizes the “Top Ten” selection, but also interesting initiatives to impulse innovation, such as the “Chemistry Entrepreneurship” project, promoted by IUPAC’s Committee on Chemistry and Industry. This initiative, which started as a series of webinars, has grown into a bigger program to bridge the gap between academia and industry, counting on the connections created by entrepreneurs.^3^ Creating a culture of entrepreneurship has demonstrated different benefits for green transition, such as sustained business growth and employment, as well as successful long-term solutions for issues such as climate crises. Similarly, promoting entrepreneurship within the chemical sciences, IUPAC seeks to expedite the application of research to address pressing global challenges in energy, the environment, and health, underscoring the importance of bringing scientific innovations to the marketplace.

## The “Next Big Thing”

Often, chemical discoveries stay secluded, published in paywalled papers, or presented in small and specialized symposia. The “secrecy” of some discoveries limits technology transfer and applications, creating silos that limit progress instead of promoting access to interesting insights and information exchange. Many authors have noted the importance of interdisciplinary research in complex fields, such as climate change and human health, in which breaking down silos could become key to creating a culture of cross-collaboration that cultivates curiosity and a more holistic approach to scientific exploration. The IUPAC “Top Ten” follows the same spirit. A group of experts selected by IUPAC carefully curates a catalogue of emerging technologies from a pool of public nominations submitted by chemists and researchers worldwide. The idea behind this is to highlight promising discoveries hovering between the very early stages of laboratory curiosities to industrial success,^4^ in the spirit of attracting attention and fostering inspiration across stakeholders to eventually “bridge the gap” between commercialization and global applications. For example, the 2024 selection highlighted the discovery of MXenes, which are marvelous materials that stem from the isolation of graphene through mechanical exfoliation of graphite into atomically thin layers. This technique triggered the exploration of other “layered materials”, among them MXenes—inorganic compounds including carbides, nitrides, and carbonitrides with reported revolutionary applications in energy storage, electronics, telecommunications, and even environmental remediation, including some uses in water filtration and purification. Although still far from commercial applications, the buzz around MXenes and layered materials has attracted the attention of global companies, such as Intel and Samsung, which could provide commercial applications sooner than expected. On a different scale, the 2024 “Top Ten” selection also explored concepts that could completely reshape the chemical industry, such as the electrification of key reactions in the nitrogen cycle.^3^ A switch between thermal chemistry, which is often linked to countless carbon emissions, and electrochemistry, powered by renewable energy sources such as solar, wind, hydropower, and geothermal, could cut the climate impact of the industry, as well as enable delocalization—an interesting strategy to solve issues around the supply chain. Other exciting technologies in the latest list included a totally unprecedented adsorption phenomenon, “mechanisorption”, which uses molecular machines to move, adsorb, and desorb a series of substances; and hydration lubrication, another peculiar and paradoxical effect that appears in certain materials and conditions in response to mechanical stimuli such as friction, which could convey applications in biomedicine. Even if not all the selected technologies transform the current chemical landscape, the list still succeeds in creating connections across disciplines, boosting collaborations, and capturing the attention of entrepreneurs, early adopters, industry leaders, and investors.^5^ The “Top Ten” lists have included success stories like mRNA vaccines, enantioselective organocatalysis, and artificial intelligence applications in chemistry—all later recognized with the Nobel Prize in chemistry. The potential to pick “the next big thing” in chemistry is there, all backed by the trustworthiness of IUPAC.

## From Discovery to Commercial Reality: Catalyzing Chemistry Entrepreneurship

Despite significant advances in chemical discovery and the potential of many of the emerging technologies that IUPAC highlights each year, many innovations never leave the laboratory. Some suggest the problem is the lack of entrepreneurial culture in chemistry, hindered by the inherent risk of working in a start-up, the large investments needed for commercializing a new technology, and the increasingly demanding regulations. Nevertheless, entrepreneurship is key to bringing the benefits of chemistry discovery to all, as showcased by success stories such as the Oxford-Astrazeneca Covid-19 vaccine, and the idea of including electrolytes in sports drinks, which eventually sparked the creation of Gatorade. Luckily, more and more schemes support start-ups and entrepreneurship in chemistry—including new public funding agencies devoted to technology transfer across the world, such as the National Research Foundation in India, the European Innovation Council in the EU, and the new boost to the budget in science and technology recently announced by the Chinese government. Moreover, several initiatives by associations like the American Chemical Society, the Royal Society of Chemistry, and of course IUPAC, have switched the focus to further support chemistry start-ups and entrepreneurs, including policy recommendations to governments to prioritise technology transfer via more competitive tax policies, incentives to investors, accessible grants, and seed money for licensing and commercialization.

The conversion of carbon dioxide into value-added molecules—the creation of solar synthetic fuels, highlighted by IUPAC in the 2022 list^6^—has provided some interesting successful examples in entrepreneurship. For example, Dioxycle and Turnover Laboratories have secured investments to valorise CO_2_ using electrolysis to manufacture sustainable hydrocarbons, such as ethylene. Similarly, companies such as Twelve and Air Co. have attracted the attention of airlines and engineering firms because of their potential to transform captured carbon dioxide into sustainable aviation fuels (SAFs) which, although still more expensive than traditional alternatives, could reduce emissions by up to 87%, thereby making air travel more environmentally friendly and closer to meeting the demands of net-zero. These examples illustrate the dynamic landscape of the current chemical industry in which entrepreneurs and start-ups drive innovation and attract key companies to leverage advances in carbon capture, electrolysis, and catalysis to speed up sustainable solutions to pressing societal challenges.

The commercialization of chemical research (see Figure 1) faces numerous challenges, including limited funding, disparity between academic innovations and market demands, and inherent resistance to interdisciplinary collaboration. By establishing robust academia–industry linkages, dedicated entrepreneurship programs, and the promotion of partnerships with venture capital, obstacles could be transformed into opportunities for sustained innovation and long-term impact.^7^

**Figure 1:**
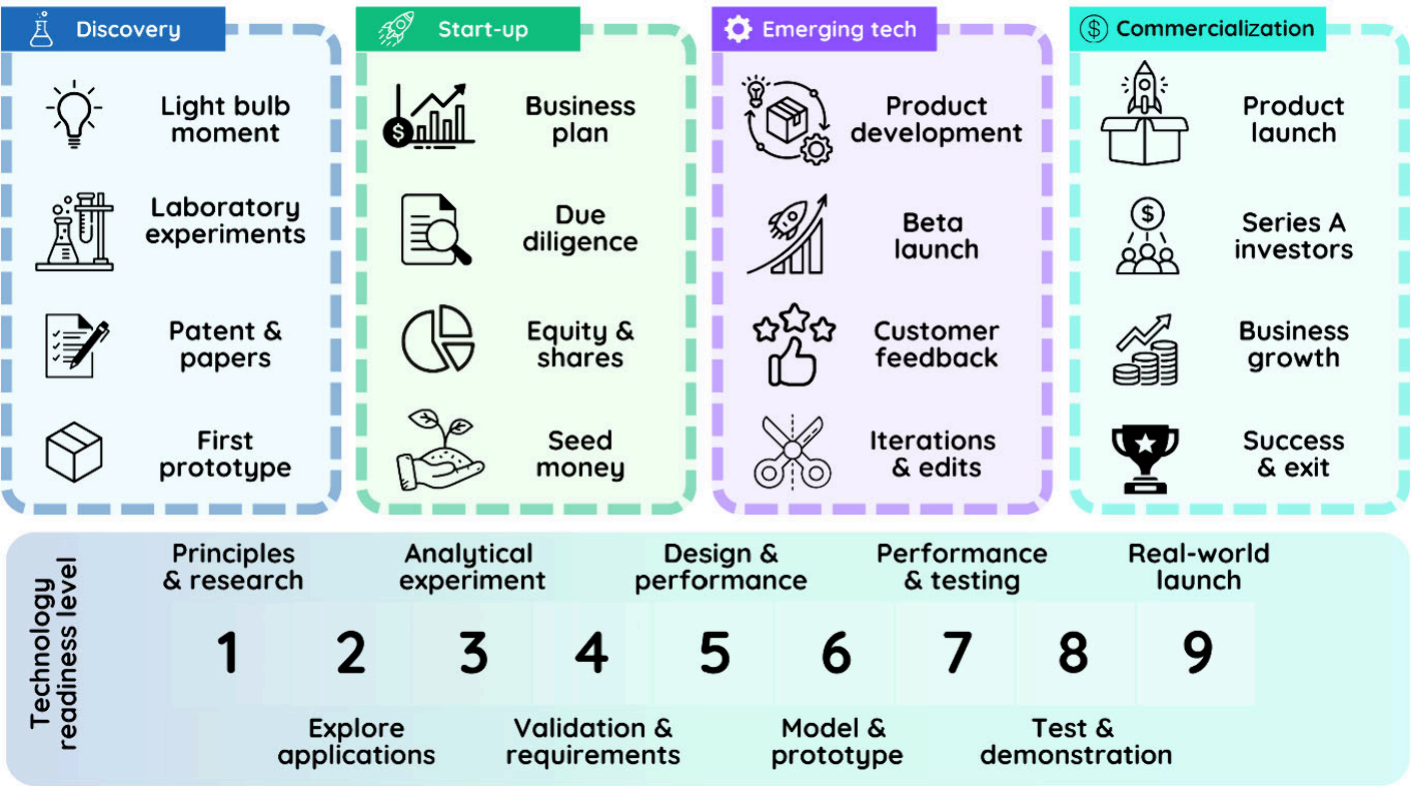
From lab to market: stages of commercialization and Technology Readiness Levels (TRLs) in chemical research.

To contribute toward this end, IUPAC has launched an international project “Chemistry Entrepreneurship” (Project 2023-012-2-022) to bridge the gap between laboratory discoveries and market applications. This project aims to empower chemists with the entrepreneurial skills necessary to transform innovative research into viable commercial products and services. As part of this initiative, IUPAC is organizing a series of webinars designed to guide chemists through the process of bringing their laboratory discoveries to the marketplace. Topics covered include identifying market needs, developing business plans, securing funding, and navigating regulatory landscapes. By participating in these sessions, chemists can gain valuable insights into the entrepreneurial journey, enabling them to effectively translate their scientific innovations into impactful market solutions.

Through the Chemistry Entrepreneurship Project and its associated webinars, IUPAC is fostering a culture of innovation and entrepreneurship within the chemical sciences, ultimately contributing to economic growth and societal advancement.

## Emerging Technologies for a Sustainable Chemical Manufacturing

The 2030 deadline for a sustainable chemical industry is rapidly approaching—just five years away. The question is: are we, chemists, truly prepared? Achieving this ambitious goal requires a comprehensive transformation across the sector. This includes developing reliable green chemistry metrics, crafting realistic roadmaps for sustainable transitions, innovating energy utilization, revising regulatory frameworks, and reimagining chemical education to embrace a broader diversity of ideas and circularity by design. The urgency is clear: *the time to act is now*.

Opportunities in digital technology present a great opportunity to accelerate this transition along the value chain. For example, artificial intelligence can help assess the toxicity of chemicals faster than current solutions, speeding up the development of drugs and pharmaceuticals, and tools such as digital twins can boost the productivity of chemical plants, simultaneously increasing safety. Additionally, digital tools could tackle the issues of silos, allowing for more efficient data management and sharing, and creating collaborative ecosystems connecting companies in different sectors. An interesting example is Catena-X, a collaborative network impulsed by leading industries in Europe and the German government, which enables secure data sharing between researchers, suppliers, and manufacturers; it not only catalyzes innovations, but also improves the reliability of end-to-end traceability, which in turn supports sustainability and avoids supply chain disruptions. Indeed, supply chains could also benefit from digital solutions and artificial intelligence, exemplified by the highlight of blockchain technology for more reproducible and traceable chemistry in the “Top Ten” list in 2021.^8^ The digitalization of the chemical industry could contribute to many aspects of the green transition, including disruptive innovations in energy sources toward replacing fossil fuels, new approaches to the design and development of molecules with sustainability, circularity, and recyclability at the heart, and more efficient logistics, prioritizing delocalized and decentralized manufacturing to maximize productivity, on-demand fabrication, and local supplies instead of critical resources.^9,10^

## Conclusion

The IUPAC “Top Ten Emerging Technologies in Chemistry” are ready to revolutionize the chemical industry, driving groundbreaking innovation, enhancing global competitiveness, and accelerating the shift toward a circular economy—an essential step in achieving net-zero carbon goals. Notably, the **digitalization of chemistry** and cutting-edge advancements in **artificial intelligence**—further underscored by the 2024 Nobel Prizes in both Physics and Chemistry—are reshaping the landscape of resource tracking, waste management, and energy efficiency. These transformative technologies are redefining industrial processes, ensuring they align seamlessly with sustainability and the future of green chemistry.

To maintain industrial competitiveness while meeting the Green Deal objectives, we must actively integrate disruptive technologies into the value chain. This not only slashes emissions, but also fuels economic growth by creating more efficient, adaptive, and resilient industrial processes. Startups and industry pioneers must champion the commercialization of cutting-edge solutions, ensuring that AI-driven resource optimization, next-generation catalysts, and digital chemistry tools become integral to sustainable manufacturing. That is where chemistry entrepreneurship can play a role and how IUPAC is also contributing to not only highlighting and giving visibility to the most promising technologies, but also providing the resources and educational materials that will enable the transition of these technologies from the lab to the market.
